# Understanding the precipitation mechanism in pentavalent vanadium electrolytes through deep learning potential molecular dynamics

**DOI:** 10.1039/d6sc02403c

**Published:** 2026-04-27

**Authors:** Chenkai Mu, Chenbo Zhan, Tianyu Li, Xianfeng Li

**Affiliations:** a Division of Energy Storage, Dalian National Laboratory for Clean Energy, Dalian Institute of Chemical Physics, Chinese Academy of Sciences Dalian 116023 China lixianfeng@dicp.ac.cn litianyu@dicp.ac.cn; b Key Laboratory of Long-Duration and Large-Scale Energy Storage, Chinese Academy of Sciences Dalian 116023 China; c School of Chemistry and Chemical Engineering, University of Chinese Academy of Sciences Beijing 100049 China

## Abstract

Vanadium flow batteries (VFBs) represent one of the promising options for large-scale energy storage, yet the precipitation of pentavalent (V(v)) species under elevated temperature and high state-of-charge severely limits energy density and cycle life. However, due to the lack of approaches capable of tracking the liquid-to-solid transition at atomic resolution, the mechanism underlying this precipitation has remained elusive. Here, we employ deep potential molecular dynamics (DPMD) to investigate the precipitation process in vanadium electrolytes. A high-accuracy deep potential model, with energy errors below 1 meV and force errors below 60 meV Å^−1^, was developed through active learning. And complete transformation of V(v) from hydrated species to vanadium oxide precipitates was simulated. The results reveal that precipitation proceeds *via* hydroxyl dehydration-transformation following an SN2-type pathway with an activation barrier of approximately 40 kJ mol^−1^. Based on these mechanistic insights, an anion coordination strategy was proposed to suppress precipitation. Experimental validation confirmed that strongly coordinating anions such as phosphate and arsenate extend precipitation onset from 10 hours to 150–200 hours at 50 °C. This study elucidates the precipitation mechanism and provides guidance for electrolyte formulation optimization in VFBs.

## Abstract 2

Deep potential molecular dynamics was employed to reveal the atomic-level precipitation mechanism of pentavalent vanadium in VFB electrolytes.

## Graphical abstract

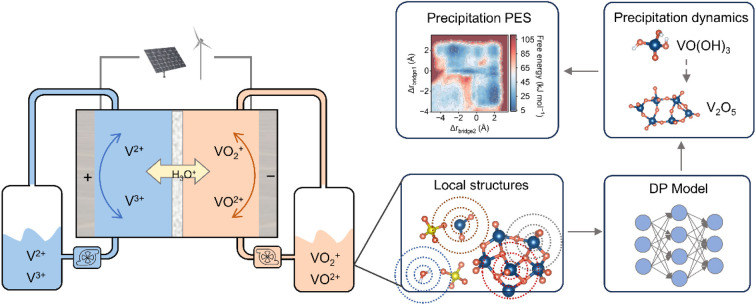

## Introduction

**Fig. 1:**
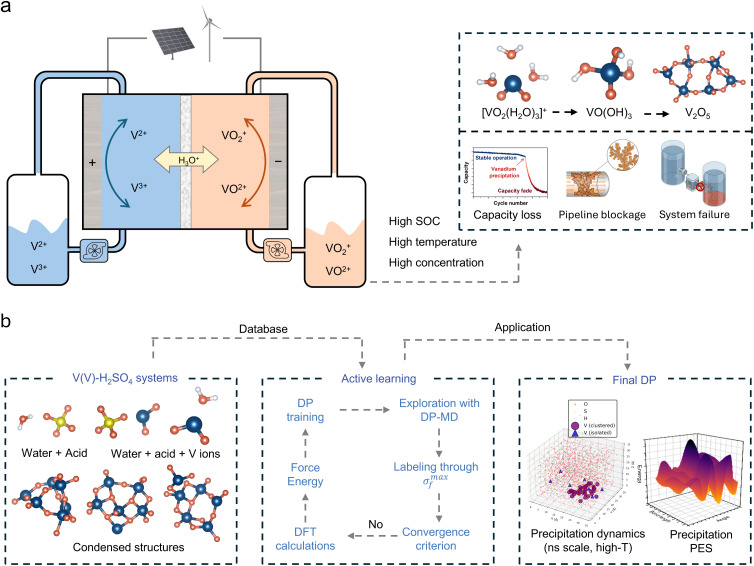
Research background and workflow of training DP. (a) Schematic of VFBs operation and positive electrolyte precipitation. The battery structure employs V^2+^/V^3+^ and VO_2_^+^/VO^2+^ redox couples at negative and positive electrodes respectively. Under high SOC, elevated temperatures, and high concentration, V(v) species transform from hydrated [VO_2_(H_2_O)_3_]^+^ through hydroxylated intermediate VO(OH)_3_ to V_2_O_5_ precipitate, causing capacity loss, pipeline blockage, and system failure. (b) Iterative development workflow for deep potential model construction through active learning, including exploratory simulation, uncertainty-based structure selection, DFT labeling, and model retraining until convergence.

Vanadium flow batteries (VFBs) have attracted widespread attention as promising technologies for large-scale energy storage owing to their high efficiency, long cycle life, and high safety.^1–4^ However, precipitation of pentavalent vanadium (V(v)) ions from the positive electrolyte at elevated temperatures and high state-of-charge (SOC) severely limits energy density and long-term operational stability, potentially causing pipeline blockage and system failure (Fig. 1a).^5–7^ Although engineering strategies such as thermal management, increasing sulfuric acid concentration, and the use of stabilizing additives can suppress the precipitation, the underlying mechanism remains poorly understood.^8,9^ This knowledge gap arises largely from the lack of techniques capable of tracking the solution-to-solid phase transition at atomic resolution, thus hindering the rational optimization of electrolyte formulations and the design of effective precipitation inhibition strategies.^10–12^

In our previous work, we employed *ab initio* molecular dynamics (AIMD) combined with *in situ* liquid time of flight secondary ion mass spectrometry (ToF-SIMS) to elucidate the stepwise deprotonation mechanism of V(v) species in aqueous electrolytes.^13^ However, the complete pathway from hydroxylated intermediates to solid V_2_O_5_ precipitates remains unresolved due to the spatiotemporal limitations of existing methods. Investigating such precipitation reactions in aqueous electrolytes is inherently challenging, as the underlying elementary steps occur at atomic-scale spatial dimensions and femtosecond-to-picosecond temporal scales that are difficult to access with current experimental techniques and computational methods.^14–16^ While techniques such as *in situ* X-ray absorption spectroscopy (XAS), Raman spectroscopy, and nuclear magnetic resonance (NMR) have provided valuable insights into speciation and local structure, they lack the resolution to fully capture the dynamic evolution of precipitation in complex aqueous electrolytes.^17–19^ Computational simulations offer atomic-level insights that are inaccessible to experimental techniques, but traditional methods face a fundamental trade-off between accuracy and scale.^20^ AIMD enables the investigation of chemical reactions with quantum mechanical accuracy at the density functional theory (DFT) level for systems containing hundreds of atoms over picosecond timescales.^21^ However, the prohibitive computational cost renders large-scale, long-timescale simulations impractical.^22,23^ Classical molecular dynamics (MD) enables simulations of systems containing thousands to millions of atoms over nanosecond-to-microsecond timescales but relies on predefined force fields that are incapable of describing chemical bond breaking and formation, rendering it unsuitable for studying complex reactions involving hydrolysis and polycondensation.^24–26^ To address these limitations, the deep potential (DP) method has emerged as a powerful solution.^27,28^ By training deep neural networks on first-principles data, DP achieves near-quantum mechanical accuracy while maintaining the efficiency of classical force fields, enabling simulations of thousands to tens of thousands of atoms over hundreds of nanoseconds.^29^ Deep potential molecular dynamics (DPMD) has been successfully applied to diverse chemical systems, including metal phase transitions and defect evolution,^30^ dynamics of growing carbon nanotubes,^31^ and catalytic reaction mechanisms.^32^ Its capability to accurately describe chemical reaction pathways and phase transition processes makes it an ideal tool for tracking the complete evolution from solvated ions to solid precipitates.^33–35^

This work employs DPMD to investigate the precipitation process in electrolyte systems, providing atomic-level insights into the formation of solid precipitates from V(v) solutions. An active learning approach was employed to construct a training dataset including hydrated ions, hydroxylated intermediates, and various aggregated structures, enabling the development of a high-accuracy DP model. Using this model, we successfully simulated the complete transformation of V(v) ions from solvated ions to solid V(v) precipitates, revealing the nucleation mechanism and microscopic pathway at atomic resolution. The molecular mechanism was elucidated through quantitative tracking of key bond evolution, free energy surface calculations along reaction pathways, and systematic investigation of temperature and concentration effects on precipitation. Beyond explaining the precipitation mechanism, this study demonstrates the capability of deep learning potentials for resolving complex solid–liquid phase transitions in electrolyte systems, providing both theoretical insights and a practical framework for investigating solution-phase reactions from simulation to experimental validation.

## Results and discussion

**Fig. 2:**
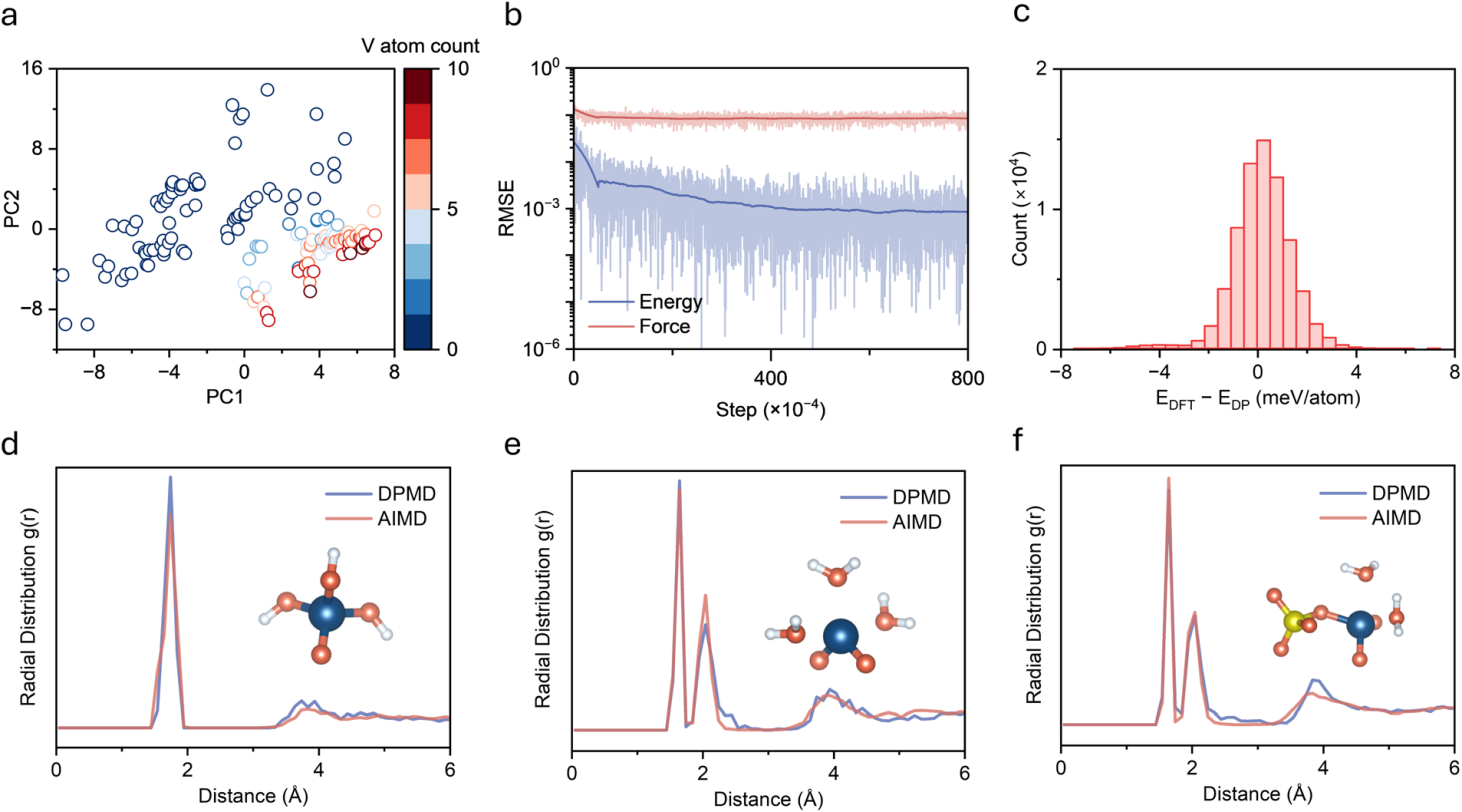
Deep potential model validation. (a) Principal component analysis showing structural diversity of training dataset with colors indicating vanadium atom count. (b) Root mean square error evolution for energy and force during training. (c) Error distribution histogram between DFT and DP predicted energies. (d–f) Radial distribution function comparison between DPMD and AIMD for (d) VO(OH)_3_, (e) [VO_2_(H_2_O)_3_]^+^, and (f) [VO_2_(H_2_O)_2_SO_4_]^−^, with insets showing representative local coordination structures.

An accurate description of the complete precipitation reaction pathway from solution to solid precipitate in V(v) electrolyte systems requires construction of a potential function with quantum mechanical accuracy covering all relevant chemical environments. An active learning strategy was employed to systematically generate training data, owing to the fact that this system involves interconversion among multiple chemical species including hydrated vanadium oxo ions, hydroxylated species with varying protonation degrees, and aggregated structures with various polymerization degrees. Model development followed an iterative optimization workflow (Fig. 1b). An initial database was constructed from vanadium species with different numbers of water and sulfuric acid molecules (Fig. S1), which was subsequently refined through successive rounds of active learning. During each round of DPMD simulation, model prediction uncertainty was monitored to automatically identify novel structures, which were then labeled *via* DFT calculations and added to the training set for continuous model improvement. The active learning protocol captures transition-state geometries through NVT simulations at 600–800 K, with uncertainty-based selection (force deviation: 0.16–0.50 eV Å^−1^). After multiple iterations, a final dataset containing 76 172 configurations was established. The V–V distance distribution across the entire training dataset (Fig. S2) confirms coverage of the complete range from isolated monomers to closely aggregated states. To assess the structural diversity of the training dataset, principal component analysis (PCA) was performed on all configurations using comprehensive structural descriptors including geometric features (radius of gyration, interatomic distances), element-specific features (V–O bond lengths, coordination numbers, V–V distances), and bonding characteristics (short/long V–O bonds). PC1 primarily captures the degree of vanadium aggregation (dominated by average V–V distance, coordination number, and V–O bond length, Fig. S3), while PC2 reflects variations in local coordination environment. The distribution of training set configurations in PC1–PC2 space shows a continuous spread across different vanadium concentrations without obvious separation (Fig. 2a), indicating successful sampling of the complete structural evolution from solvated V(v) ions to vanadium oxide species and providing a robust foundation for accurately describing the precipitation process.

The final DP model was trained on the above-mentioned dataset. Training errors for energy and force converge after 8 million training steps, with energy errors below 1 meV and force errors below 60 meV Å^−1^ (Fig. 2b). Validation on the complete dataset shows that over 80% of the DP predictions deviate from DFT values by less than ±1 meV per atom (Fig. 2c), demonstrating sufficient accuracy for reliable description of energy changes during chemical reactions. Force predictions on the full dataset show excellent linear correlation with DFT calculations in all three Cartesian directions (Fig. S4), indicating reliable prediction of interatomic forces across the entire configuration space. To evaluate model quality and generalization capability, radial distribution functions (RDFs) from DPMD and AIMD simulations were compared for three pentavalent vanadium compounds, including VO(OH)_3_, hydrated [VO_2_(H_2_O)_3_]^+^, and [VO_2_(H_2_O)_2_(SO_4_)]^−^ complexes. The RDFs from both methods are in good agreement (Fig. 2d–f), showing consistent peak positions and intensities in the major coordination shells and accurate representation of outer solvation shell structures. These results demonstrate that the developed DP model not only achieves high accurate in energy but also faithfully captures local structural features across diverse chemical environments, enabling large-scale, long-timescale simulations.

**Fig. 3:**
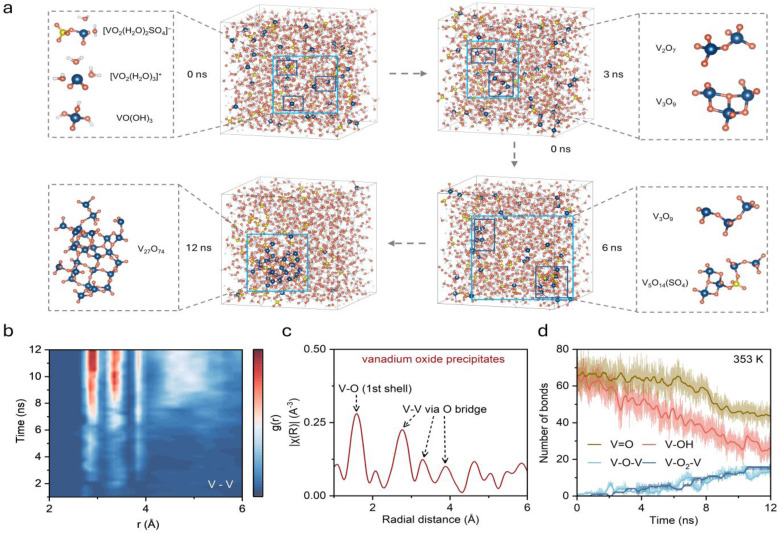
Kinetic evolution of pentavalent vanadium precipitation. (a) Representative snapshots from 12 ns DPMD simulation showing the transformation of V(v) species from monomers to polynuclear clusters, with magnified views of local structural evolution. (b) Time-resolved V–V RDF heatmap showing increasing intensity in the 2–6 Å range, indicating formation of oxygen-bridged vanadium species. (c) Fourier-transformed EXAFS spectrum |*χ*(*R*)| of V(v) precipitate. (d) Evolution of different coordination bond types over time, with V=O and V–OH decrease while V–O–V and V–O_2_–V increase.

To track the complete evolution from solvated ions to solid precipitates, DPMD simulations exceeding 12 ns were performed on a system containing 2 M VO_2_^+^, 2 M sulfuric acid, and water molecules at 353 K (Table S1). The simulation temperature of 353 K was chosen to access complete precipitation within tractable timescales, following the standard practice of using elevated temperatures to accelerate rare-event kinetics in MD simulations. The transformation from dispersed V(v) ions to network-like V_2_O_5_ structures was successfully captured. Representative structural evolution snapshots (Fig. 3a) show that V(v) ions initially exist as isolated monomers dispersed in solution, rapidly transforming from hydrated to hydroxylated structures consistent with previous observations (Fig. S5). As simulation progresses, adjacent VO(OH)_3_ units begin forming V–O–V bridge connections through shared oxygen atoms. These V–O–V bridges initially form as small oligomers (dimers and trimers), but over nanosecond timescales, these oligomers continue interconnecting to develop into larger vanadium oxide condensed structures. Cluster size analysis (Fig. S6) shows that by ∼10 ns, over 70% of V(v) ions have aggregated into polynuclear clusters. By late simulation stages, a three-dimensional network structure formed by multiple vanadium atoms connected through oxygen bridges becomes clearly visible—the characteristic structure of solid V_2_O_5_ precipitate. Sulfuric acid and water molecules remain uniformly distributed throughout, consistent with V_2_O_5_ precipitation from the liquid phase. Local structures analysis reveals a gradual evolution from isolated tetrahedral vanadium units to interconnected networks sharing vertices and edges, consistent with the main structure in solid vanadium oxides (Fig. S7).

Quantitative description of this precipitation process was achieved through analyzing the time evolution V–V RDF. The time-resolved RDF (Fig. 3b) shows continuous increase in V–V correlation intensity in the 2–4 Å range, reflecting the progressive formation of oxygen-bridged V–V pairs and increasing aggregation. This distance is characteristic of vanadium atoms connected through bridging oxygen in oligomeric and polynuclear clusters. Comparison with Fourier-transformed extended X-ray absorption fine structure (EXAFS) spectra |*χ*(*R*)| from electrolyte precipitates (Fig. 3c) shows good agreement in major peak positions, confirming that the simulation accurately captures structural evolution from solvated ions to solid precipitate states. Coordination bond population analysis provides direct insights into the reaction mechanism. Four key coordination types were tracked throughout the simulation (Fig. 3d), including V=O, V–OH, V–O–V, and V–O_2_–V. Based on the evolution of average bond number in the system, the precipitation process can be divided into three stages. In the initial stage (0–6 ns), V–OH decreases sharply from approximately 65 to 40 while V–O–V bridges begin forming from 0 to 6, with V=O remaining stable around 70, indicating hydroxyl transformation dominates. During the intermediate stage (6–10 ns), V–O–V formation accelerates to about 10 and V=O begins declining. In the final stage (>10 ns), extensive bridging develops with V–O–V and V–O_2_–V reaching approximately 15. This stepwise evolution reveals that precipitation proceeds through hydroxyl transformation followed by vanadyl incorporation, providing quantitative evidence for designing targeted inhibition strategies.

To guide the design of precipitation inhibition strategies, the influence of sulfuric acid concentration and temperature on precipitation dynamics was systematically investigated. The bridge formation rate, defined as the total number of V–O–V and V–O_2_–V bonds formed per nanosecond, was used to quantify precipitation kinetics. Increasing sulfuric acid concentration significantly inhibits precipitation. The bridge formation rate decreases from 2.33 to 0.83 bridges per ns when sulfuric acid concentration increases from 2 M to 4 M (Fig. S8). This suppression correlates with enhanced sulfate coordination, as V–O–S bonds increase from approximately 7 to 15 (Fig. S9), indicating that sulfate ions competitively inhibit V–O–V bridge formation. Structural analysis shows that vanadium species remain predominantly dispersed, with over 70% of vanadium atoms existing as monomers or dimers after 16 ns and no clusters exceeding 10 vanadium atoms observed (Fig. S10). This contrasts sharply with the extended polynuclear clusters formed under 2 M conditions. In contrast, elevated temperature significantly accelerates precipitation kinetics. The bridge formation rate increases from 0.25 bridges per ns at 298 K to 0.75 bridges per ns at 323 K and 2.33 bridges per ns at 353 K, representing a nearly tenfold enhancement (Fig. S11). At 298 K, vanadium species remain predominantly as isolated oligomers with minimal bridging oxygen formation even after 20 ns (Fig. S12). At 323 K, the bridge formation rate increases to 0.75 bridges per ns, showing approximately threefold enhancement compared to room temperature (Fig. S13). These results provide molecular-level understanding of the experimental observations that high acid concentration stabilizes the electrolyte whereas elevated temperature accelerates precipitation, offering quantitative guidance for thermal management and electrolyte optimization.

**Fig. 4:**
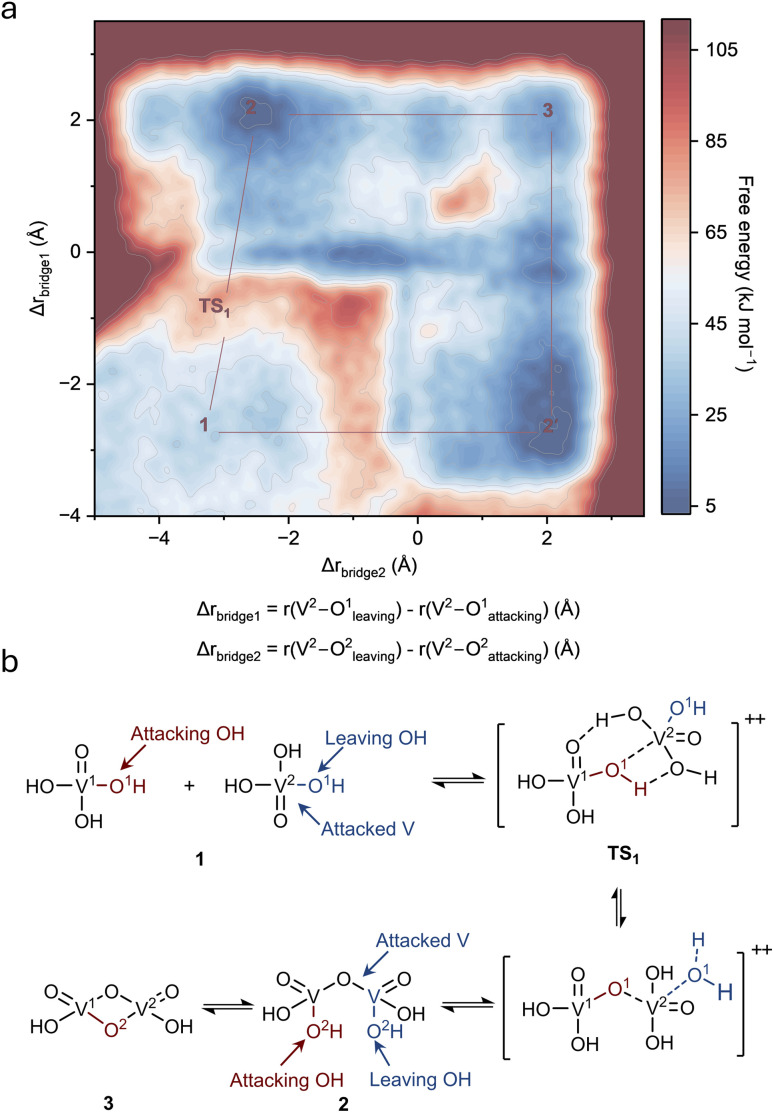
Potential energy surface analysis of free energy for dehydration transformation reaction. (a) Two-dimensional free energy surface as a function of reaction coordinates Δ*r*_bridge1_ and Δ*r*_bridge2_, tracking the progress of dehydration-transformation. State 1 represents two isolated VO(OH)3 units, TS1 represents the transition state with approximately 40 kJ mol^−1^ energy barrier. State 2 shows a dimer with single V–O–V bridge, and State 3 shows a dimer with double V–O^2^–V bridge. (b) Schematic of the SN2 mechanism between V^1^O(OH)3 (attacking unit) and V^2^O(OH)3 (attacked unit). Red indicates the attacking hydroxyl from V^1^ and blue indicates the leaving hydroxyl from V^2^. The concerted substitution forms oxygen-bridged vanadium structures with water elimination.

The molecular-level reaction mechanism was further investigated to understand the thermodynamics and kinetics of the reaction. Trajectory analysis revealed that the precipitation proceeds through dehydration-transformation reactions between adjacent VO(OH)_3_ units *via* a bimolecular nucleophilic substitution (SN2) mechanism. In this process, a hydroxyl group from one VO(OH)_3_ (designated as V^1^O(OH)_3_, the attacking unit) acts as a nucleophile to attack the vanadium center of a neighboring unit (designated as V^2^O(OH)_3_, the attacked unit), while a hydroxyl ligand from the attacked unit departs as water, forming a V–O–V bridge. This SN2-type mechanism provides atomistic resolution of the elementary steps and transition-state geometry that are inaccessible to the classical olation and oxolation descriptions derived from bulk spectroscopic characterization. To quantify the reaction energetics, the free energy surface was calculated using two reaction coordinates that track the progress of bridge formation (Fig. 4a). For the first V–O–V bridge formation, we define Δ*r*_bridge1_ = *r*(V^2^ – O^1^_leaving_) – *r*(V^2^ – O^1^_attacking_), where *r*(V^2^ – O^1^_leaving_) is the distance between the departing hydroxyl oxygen (originally coordinated to V^2^) and V^2^, and *r*(V^2^ – O^1^_attacking_) is the distance between the attacking oxygen (originally coordinated to V^1^) and V^2^. Negative values of Δ*r*_bridge1_ indicate the reactant state with the attacking oxygen far from vanadium, Δ*r*_bridge1_ ≈ 0 corresponds to the transition state where both oxygens are approximately equidistant from the vanadium center, and positive values represent the product state with the leaving group departed. Similarly, Δ*r*_bridge2_ describes the formation of the second V–O–V bridge through an identical SN2 mechanism. Fig. 4a shows that the reaction initiates from State 1, where two isolated VO(OH)_3_ units exist at a local energy minimum. As the hydroxyl oxygen from V^1^O(OH)_3_ approaches the vanadium center of V^2^O(OH)_3_, the system overcomes an energy barrier of approximately 40 kJ mol^−1^ to reach transition state TS_1_. At TS_1_, the attacking oxygen forms weak interactions with the vanadium while the departing hydroxyl prepares to leave, exhibiting five-coordinate geometry characteristic of SN2 transition states. After crossing TS_1_, a dimer linked through a single V^1^–O–V^2^ bridge (State 2) forms with slightly lower free energy than State 1, indicating thermodynamic favourability. State 2 can further transform to a more stable double oxygen bridge structure (State 3) following an identical SN2 mechanism. The reaction pathway (Fig. 4b) illustrates this mechanism with V^1^O(OH)_3_ and V_2_O(OH)_3_ clearly labelled, the attacking hydroxyl marked in red, and the leaving hydroxyl marked in blue. The red hydrogen present at TS_1_ is released into the bulk aqueous solution following bridge formation, while the blue hydrogen appearing in State 2 originates from proton transfer from the surrounding solvent to the oxygen on V^2^, which subsequently serves as the leaving group for the next SN2 step. The overall precipitation consists of cascading dehydration-transformation reactions, with each step involving coordination changes and progressive expansion of the V–O–V network.

**Fig. 5:**
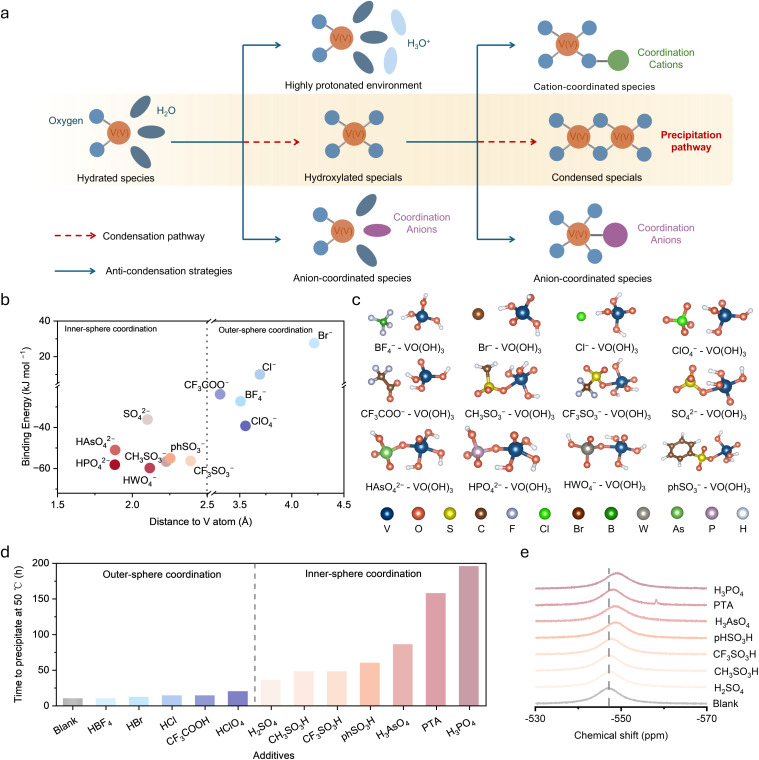
Anion coordination inhibition strategy. (a) Transformation pathway from solvated to precipitated species and strategies to overcome precipitation *via* high protonation environment and anion coordination. (b) Binding energy *versus* V-anion distance for 12 anions with VO(OH)_3_, with dashed line separating inner-sphere (<2.5 Å) and outer-sphere (>2.5 Å) coordination regions. (c) Optimized structures of anion-VO(OH)_3_ complexes showing different coordination modes. (d) Experimentally measured precipitation time at 50 °C with different acid additives. (e) ^51^V NMR chemical shift spectra showing significant shifts with inner-sphere coordinating anions.

We proposed precipitation inhibition strategies targeting different stages of the pathway based on the mechanism and summarized in Fig. 5a. The complete transformation from solvated ions to precipitates occurs *via* two key steps. Under elevated temperature and high V(v) concentration, hydrated VO_2_^+^ first converts to hydroxylated VO(OH)_3_ species, which subsequently undergo dehydration-transformation to form condensed networks. The first step is mainly governed by solution acidity and coordination environment. High proton concentrations stabilize hydrated species by suppressing deprotonation of coordinated water, while strongly coordinating anions can directly bind to VO_2_^+^ to inhibit hydroxylation, as previously reported.^13^ The second step is the focus of the present study, with VO(OH)_3_ serving as the key intermediate in the precipitation pathway. Ligand coordination strategies can effectively suppress the dehydration-transformation reaction by occupying vanadium coordination sites or disrupting intermolecular hydroxyl interactions in TS_1_, thereby sterically preventing the approach of adjacent VO(OH)_3_ molecules and hindering dehydration-transformation. Both anionic and cationic species have been reported to stabilize V(v) electrolytes through such coordination mechanisms.^9,36–39^

To investigate the coordination affinity of different anions for VO(OH)_3_, we systematically computed the binding energies of 12 anions with VO(OH)_3_. DFT structure optimization reveals that anions either spontaneously form stable coordinate complexes with VO(OH)_3_ or move away, depending on their interaction strength with VO(OH)_3_. Plotting binding energy against the optimized V-anion distance (Fig. 5b) enables classification of anions into two categories based on coordination distance. Inner-sphere coordinating anions can directly enter the first vanadium coordination shell, forming strong coordinate bonds with coordination distances typically less than 2.5 Å, including oxoanions such as phosphate, arsenate, and tungstate. These anions show binding energies of −40 to −60 kJ mol^−1^ with VO(OH)_3_, indicating very strong coordination. Outer-sphere coordinating anions interact with VO(OH)_3_ primarily through electrostatic interactions and hydrogen bonding, with coordination distances typically greater than 3.5 Å, including tetrafluoroborate, bromide, and chloride, showing relatively weak binding energies. Optimized structures of representative anion-VO(OH)_3_ complexes (Fig. 5c) clearly reveal that inner-sphere anions directly occupying vanadium coordination sites, whereas outer-sphere anions remain in the outer solvation shell.

Guided by the computational results, we experimentally validated the strategy of anion coordination by adding different anion additives to pentavalent vanadium electrolytes and monitoring the time to precipitation onset at 50 °C. The experimental results (Fig. 5d) are highly consistent with theoretical predictions. Electrolytes with strong inner-sphere coordinating anions such as phosphoric acid, phosphotungstic acid, and arsenic acid remained stable for 150–200 hours without precipitation, demonstrating effective suppression of precipitation. In contrast, electrolytes with outer-sphere coordinating anions such as tetrafluoroboric acid, hydrobromic acid, and hydrochloric acid showed precipitation within hours to tens of hours, with limited improvement compared to blank samples. This clear distinction between the two categories of anions confirms that coordination strength is the key factor determining inhibition effectiveness. To further verify the presence of coordination interactions, we performed ^51^V NMR spectroscopy to probe the vanadium coordination environment. The spectra (Fig. 5e) show that adding inner-sphere coordinating anions induces significant chemical shift changes, providing direct evidence that these anions modify the vanadium coordination environment through strong binding. In contrast, outer-sphere coordinating anions induce negligible chemical shifts (Fig. S14), consistent with their inability to form stable inner-sphere coordination. These experimental results validate the accuracy of computational predictions and demonstrate that stabilizing solution-phase pentavalent vanadium species through strong anion coordination is an effective and practically viable strategy for inhibiting precipitation.

## Conclusions

This study employs DPMD to reveal the complete mechanism of pentavalent vanadium precipitation in VFB electrolytes at atomic resolution. A high-accuracy deep potential model was developed and used to simulate the dynamic evolution from hydrated ions to solid V(v) precipitates over 12 ns. The results demonstrate that precipitation proceeds through an SN2-type hydroxyl dehydration-transformation mechanism with an activation barrier of approximately 40 kJ mol^−1^. The effects of temperature and sulfuric acid concentration on precipitation kinetics were quantitatively elucidated, providing molecular-level explanations for experimental observations. Based on these mechanistic insights, an anion coordination strategy was proposed to inhibit precipitation. Experimental validation confirmed the theoretical predictions, with strongly coordinating anions such as phosphate and arsenate extending precipitation time to 150–200 hours at 50 °C, while weakly coordinating anions showed limited effectiveness. This work provides molecular-level guidance for electrolyte formulation optimization in VFBs and demonstrates the capability of DP for investigating complex solution-phase reactions in energy storage systems.

## Author contributions

Chenkai Mu: conceptualization, methodology, investigation, formal analysis, data curation, visualization, writing – original draft, writing – review & editing. Chenbo Zhan: investigation, writing – review & editing. Tianyu Li: conceptualization, supervision, funding acquisition, resources, project administration, writing – review & editing. Xianfeng Li: conceptualization, supervision, funding acquisition, resources, project administration, writing – review & editing.

## Conflicts of interest

There are no conflicts to declare.

## Supplementary Material

SC-OLF-D6SC02403C-s001

## Data Availability

The authors declare that all the relevant data are available from the corresponding author upon request. The deep potential model training dataset, including all DFT-labeled configurations generated through active learning, along with the inputs used to label data with CP2K have been deposited in a Zenodo repository under https://doi.org/10.5281/zenodo.19066315. All experimental and computational data supporting the findings of this study are provided in the supplementary information (SI). Supplementary information: computational methods, experimental procedures, Fig. S1–S14, and Table S1. See DOI: https://doi.org/10.1039/d6sc02403c.
