# Supplementary material for: Understanding the precipitation mechanism in pentavalent vanadium electrolytes through deep learning potential molecular dynamics
Source: Chem Sci. 2026 Apr 27;17(23):11403–11. doi: 10.1039/d6sc02403c (PMC13134457; doi:10.1039/d6sc02403c)
Supplement: SC-017-D6SC02403C-s001 [file SC-017-D6SC02403C-s001.pdf]

Supplementary Information (SI) for Chemical Science.
This journal is © The Royal Society of Chemistry 2026

Supplementary Information for

Understanding the Precipitation Reaction in Pentavalent Vanadium Electrolytes through Deep Learning Potential Molecular Dynamics

Chenkai Mu [a, c], Chenbo Zhan [a, c], Tianyu Li [a, b, *], Xianfeng Li [a, b, *]

Affiliations:

[a] *Division of Energy Storage, Dalian National Laboratory for Clean Energy, Dalian Institute of Chemical Physics, Chinese Academy of Sciences, Dalian 116023, China*

[b] *Key Laboratory of Long-Duration and Large-Scale Energy Storage, Chinese Academy of Sciences, Dalian 116023, China*

[c] *School of Chemistry and Chemical Engineering, University of Chinese Academy of Sciences, Beijing 100049, China*

[*]Corresponding author. Email: litianyu@dicp.ac.cn, lixianfeng@dicp.ac.cn

Materials and Methods

Initial Dataset Generation

The initial training dataset was constructed using ab initio molecular dynamics (AIMD) simulations performed with CP2K version 2024.2[1]. The simulations employed the Perdew-Burke-Ernzerhof (PBE) exchange-correlation functional with Goedecker-Teter-Hutter (GTH) pseudopotentials and double-ζ valence plus polarization (DZVP-MOLOPT-SR-GTH) basis sets[2-4]. A plane-wave cutoff of 400 Ry was used for the auxiliary basis set expansion. Representative initial systems covering the chemical space of interest were constructed as shown in Fig.S1, including H_2SO_4-H_2O configurations with different protonation states, solvated V(V) species ranging from monomeric to oligomeric structures, and condensed structures with V-O-V bridges. AIMD simulations were performed in the NVT ensemble at 400 K for 10 ps with a time step of 1.0 fs. Temperature was controlled using the CSVR thermostat. From each trajectory, 20 structures were uniformly sampled to ensure diverse configurational coverage while maintaining computational efficiency. These sampled configurations underwent subsequent single-point energy calculations to generate high-quality reference data for deep potential training.

Single-point Energy Calculation

All sampled structures were labeled with energies and forces through single-point DFT calculations using CP2K. The computational setup employed PBE functional with DZVP-MOLOPT-SR-GTH basis sets and GTH-PBE pseudopotentials, consistent with the AIMD simulations but with enhanced accuracy. The plane-wave cutoff was increased to 600 Ry to ensure convergence of energy and force predictions. The self-consistent field convergence criterion was set to 10^{-6}. Grimme's DFT-D3 dispersion correction was included to accurately describe van der Waals interactions in the aqueous electrolyte environment. The calculated energies and atomic forces from these DFT calculations constituted the labeled dataset for training the deep potential model.

Deep Potential Model Training

DPMD simulations were enabled by training neural network-based potential energy models using the DeePMD-kit package (version 3.1.0). The deep potential model

employed the smooth edition of the Deep Potential with attention mechanism (se_atten_v2) to capture long-range correlations and multi-body interactions[5]. The descriptor was configured with a cutoff radius of 8.0 Å and a maximum of 180 neighboring atoms. The attention mechanism utilized 128 attention nodes with dot product enabled, and the axis neuron parameter was set to 16 with double precision. The fitting network consisted of three hidden layers with 240 neurons each using a residual architecture. The model was trained using an exponential decay learning rate schedule from 1.0×10^{-3} to 3.51×10^{-8} with decay steps of 5,000. The loss function weighted energy and force contributions adaptively, with energy prefactor from 0.02 to 1.0 and force prefactor from 1000 to 1.0, prioritizing force accuracy in early training stages. During active learning iterations, each model was trained for 400,000 steps. The final deep potential model was trained for 8,000,000 steps on the complete dataset.

Active Learning with DP-GEN

To systematically expand the training dataset and improve model transferability, we employed the DP-GEN2 active learning workflow[6]. The protocol consisted of 79 iterations, with each iteration training an ensemble of 4 independent deep potential models initialized with different random seeds to quantify prediction uncertainty through inter-model deviation. Each iteration performed exploratory DPMD simulations including NPT simulations at varying temperatures (230-400 K) and pressures (1.0-10,000 bar), NVT simulations at elevated temperatures (600-1000 K), and deformation simulations applying systematic box deformations. Structures from exploratory simulations were evaluated based on model deviation. Candidate structures with maximum force deviation between 0.16 and 0.50 eV/Å were selected for DFT labeling. Up to 800 structures per iteration were selected for single-point calculations. The active learning process converged when at least 90% of explored structures exhibited model deviations below the lower threshold. The final dataset comprised 76,172 labeled configurations spanning the complete precipitation pathway.

Principal Component Analysis

To assess the structural diversity and completeness of the final training dataset, principal component analysis (PCA) was performed on the 76,172 configurations using scikit-learn in Python. A comprehensive set of structural descriptors was extracted for

each configuration, including geometric features (radius of gyration, interatomic distances), element-specific features (V-O bond lengths, coordination numbers, V-V distances), and chemical bonding characteristics (short V-O bonds < 1.8 Å, long V-O bonds ≥ 1.8 Å). The feature matrix was standardized using StandardScaler before applying PCA decomposition to extract the first 10 principal components. The distribution of training configurations in the PC1-PC2 space was visualized to confirm continuous coverage across the entire structural evolution pathway from isolated monomeric species to highly aggregated polymeric networks (Fig. 2a).

Model Validation

To validate the accuracy of the trained deep potential model, we compared DPMD and AIMD simulations for three representative pentavalent vanadium species: $VO(OH)_3$, $[VO_2(H_2O)_3]^+$, and $[VO_2(H_2O)_2(SO_4)]^-$. Both DPMD and AIMD simulations were performed in the NPT ensemble at 298 K with a time step of 0.5 fs. After equilibration, production runs of 20 ps were conducted. Radial distribution functions (RDFs) were calculated from the last 10 ps of each trajectory using VMD to ensure statistical convergence and eliminate equilibration artifacts. The V-O RDFs from DPMD and AIMD simulations show nearly perfect agreement in peak positions and intensities (Fig. 2d-f), demonstrating that the deep potential model accurately reproduces the local coordination environment and solvation structure of vanadium species across different chemical states.

DPMD Simulation

All simulation systems were constructed using Packmol to randomly place vanadium ions, sulfuric acid molecules, and water molecules in cubic simulation boxes with three-dimensional periodic boundary conditions[7]. The detailed compositions of the two representative electrolyte systems are provided in Table S1. DPMD simulations were performed using LAMMPS interfaced with the trained deep potential model[8]. All simulations employed the NPT ensemble at 1 atm pressure with temperature control via the Nosé-Hoover thermostat. The integration time step was set to 0.5 fs. To investigate the effects of temperature and acid concentration on precipitation kinetics, systematic DPMD simulations were conducted under varying conditions. For the 2 M V(V) and 2 M H_2SO_4 system, simulations were performed at 298 K, 323 K, and 353 K. For the 4

M H_2SO_4 system, simulations were performed at 353 K. Bridge formation rates were obtained from the slope of linear regression applied to the cumulative bridge count versus simulation time over the window of 1–12 ns, excluding the initial 1 ns equilibration phase. All structural analyses, including radial distribution functions, coordination bond evolution, and cluster size distributions, were performed using VMD and custom Python scripts.

Free Energy Calculation

Well-tempered metadynamics simulations were performed using PLUMED interfaced with LAMMPS[9]. The simulation system consisted of two $VO(OH)_3$ units placed in a periodic simulation box with explicit water molecules and sulfuric acid. Two reaction coordinates were defined to track the progress of V-O-V bridge formation through the SN2-type mechanism. Two-dimensional well-tempered metadynamics simulations were performed with both reaction coordinates biased simultaneously. Gaussian hills with height of 5.0 kJ/mol and width (σ) of 0.1 Å were deposited every 200 steps to fill the free energy basins. A bias factor of 30 was employed to enhance sampling efficiency while maintaining accurate free energy reconstruction in the well-tempered scheme. The reaction coordinate space was explored over a grid range from -10 to +10 Å with 2000 bins. Each metadynamics simulation was run for 10 ns with a time step of 0.5 fs to ensure convergence of the free energy landscape. The two-dimensional free energy surface was reconstructed from the accumulated bias potential recorded in the HILLS file using the sum_hills utility in PLUMED.

DFT Calculations

Binding free energies were calculated using Gaussian 16[10]. Geometry optimizations and harmonic frequency calculations were performed at the B3LYP/def2SVP level of theory with Grimme's D3 dispersion correction and implicit solvation effects described by the integral equation formalism polarizable continuum model for water[11,12]. All anion complexes were initialized with the anion oxygen donor directly coordinated to the vanadium center and subjected to unconstrained geometry optimization. Weakly interacting anions spontaneously migrated to outer-sphere positions during optimization, while strongly coordinating anions retained stable inner-sphere coordination. Frequency calculations confirmed that all optimized structures were true

energy minima with no imaginary frequencies. Single-point energy calculations were performed on the optimized geometries at the B2PLYPD3/def2TZVP level of theory, providing higher accuracy for energetics. Solvation effects were treated using the SMD (Solvation Model based on Density) continuum model with water as the solvent. The binding free energy for each anion with $VO(OH)_3$ was calculated as $\Delta G = G(complex) - G(VO(OH)_3) - G(anion)$, where G represents the Gibbs free energy obtained from the frequency calculations at 298.15 K and 1 atm.

Electrolyte Preparation

Vanadium(V) electrolytes were prepared using analytical grade chemicals and ultrapure water (resistivity > 18.2 MΩ·cm). The base electrolyte was prepared by dissolving vanadium(IV) sulfate oxide hydrate ($VOSO_4 \cdot xH_2O$, 99.9%, Sigma-Aldrich) in a mixture of ultrapure water and concentrated sulfuric acid (H_2SO_4, 98%, Merck), followed by electrochemical oxidation to V(V) using a NEWARE battery testing system. The electrochemical oxidation was performed in a two-compartment cell separated by a cation exchange membrane to minimize crossover during preparation. A constant current protocol was employed to ensure complete conversion to V(V) while minimizing side reactions. The resulting V(V) electrolyte was used as the base solution for subsequent precipitation studies. For anion coordination studies, various acid additives and their corresponding sodium/ammonium salts were added to the base V(V) electrolyte at specified concentrations. The additives included hydrochloric acid (HCl, 37%, Acros), hydrobromic acid (HBr, 48%, Aladdin), tetrafluoroboric acid (HBF_4, 48%, Aladdin), perchloric acid ($HClO_4$, 70%, Sigma-Aldrich), trifluoroacetic acid (CF_3COOH, 99%, Aladdin), methanesulfonic acid (CH_3SO_3H, ≥99%, TCI), trifluoromethanesulfonic acid (CF_3SO_3H, 99%, Aladdin), phosphoric acid (H_3PO_4, 85%, Aladdin), arsenic acid (H_3AsO_4, ≥98%, Sigma-Aldrich), phosphotungstic acid hydrate ($H_3PW_{12}O_{40} \cdot xH_2O$, ≥99.9%, Aladdin), and benzenesulfonic acid ($C_6H_5SO_3H$, ≥98%, J&K Scientific). Additional sulfuric acid was also tested at varying concentrations. All electrolytes were stored in airtight, opaque containers at room temperature to prevent degradation and contamination.

Characterization

Thermal stability and precipitation onset time measurements were conducted by sealing

electrolyte aliquots in glass vials and heating in a temperature-regulated water bath. Samples were monitored visually at predetermined intervals to determine the time to precipitation onset, defined as the appearance of visible solid particles or turbidity. ^{51}V nuclear magnetic resonance spectroscopy was performed on a 400 MHz NMR spectrometer (JEOL, 9.3897 T magnetic field) with a ^{51}V resonance frequency of 105.1546 MHz. Experiments were conducted using a single-pulse sequence with a 90° pulse width. Chemical shifts were referenced to an external standard of $VOCl_3$ (0 ppm). X-ray absorption spectroscopy (XAS) measurements were conducted at beamline BL20U of the Shanghai Synchrotron Radiation Facility (SSRF), China. The V K-edge was measured and the energy was calibrated using the absorption edge of V foil. Data were recorded using a Si(111) double crystal monochromator in transmission mode. The collected XAFS data were analyzed using the Demeter software package to obtain structural parameters including coordination numbers, bond lengths, and Debye-Waller factors through EXAFS fitting.

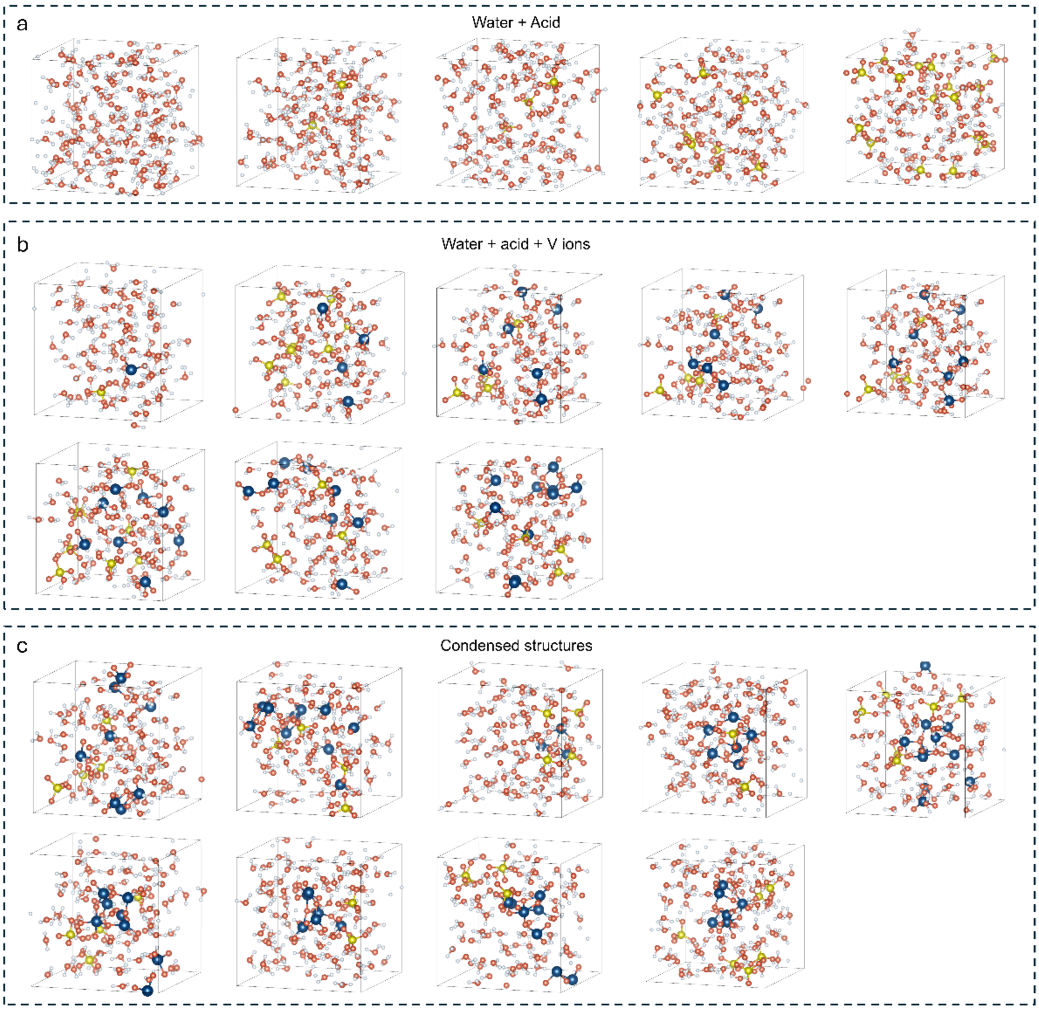

Fig. S1. Representative structures in the DP training dataset. (a) H_2SO_4-H_2O configurations showing different protonation states. (b) Solvated V(V) species ranging from monomeric to oligomeric structures. (c) Condensed structures with V-O-V bridges.

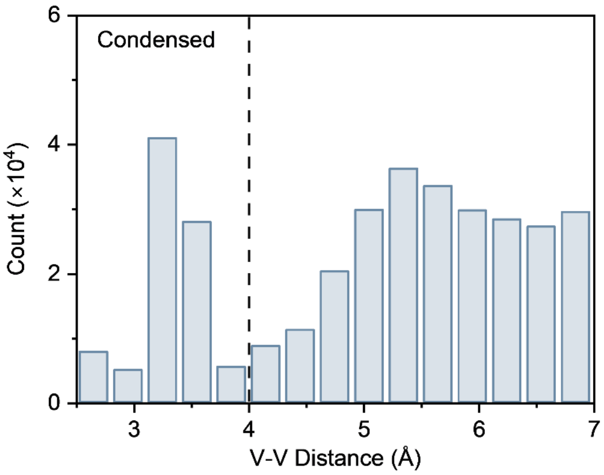

Fig. S2. V-V distance distribution in training dataset structures showing coverage from isolated monomers to condensed structures.

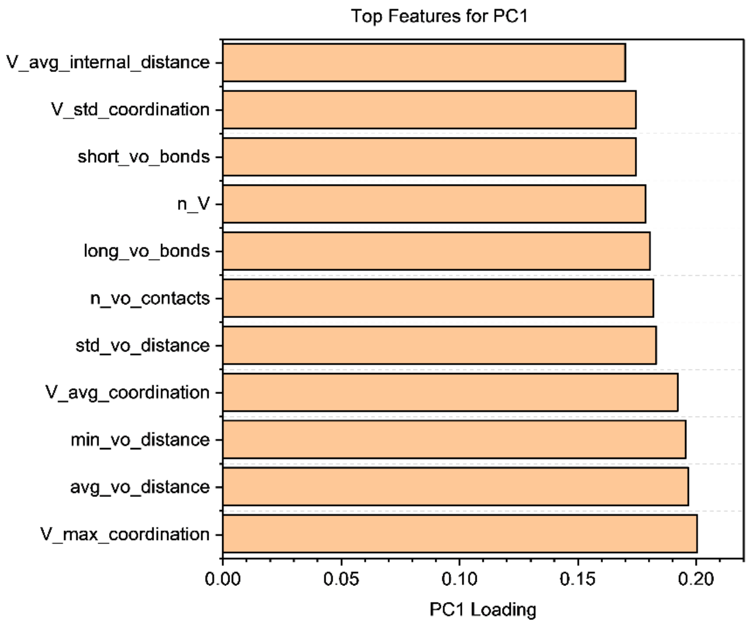

Fig. S3. Feature contribution analysis for PCA principal components showing the top 11 structural features contributing to PC1, dominated by average V-V distance, coordination number, and V-O bond lengths.

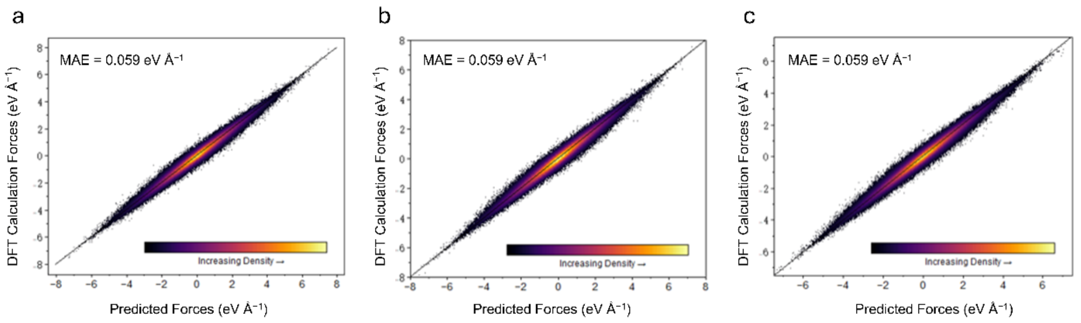

Fig. S4. Comparison of DFT-calculated and DP-predicted forces along x, y, and z axes with color intensity indicating data point density and mean absolute error of 0.059 eV $Å^{-1}$ in all directions.

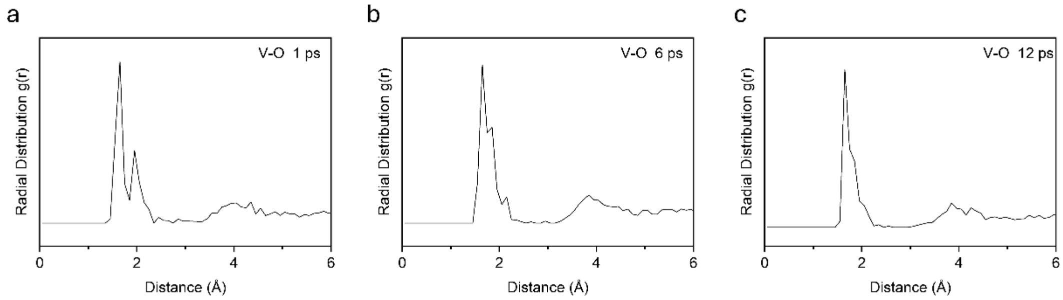

Fig. S5. V-O RDF at different simulation times. (a) 1 ps, (b) 6 ps, and (c) 12 ps. The first coordination shell peak at ~1.6 Å corresponding to V-OH bonds stabilizes rapidly, while the second shell signal from coordinated water molecules diminishes within picoseconds, demonstrating fast dehydration and hydroxylation of V(V) ions.

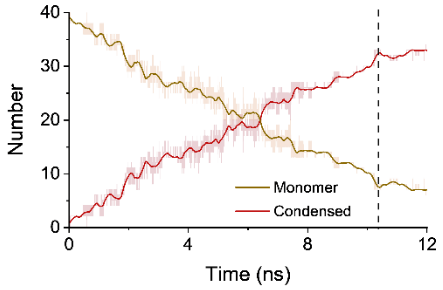

Fig. S6. Time-dependent distribution of vanadium species. Monomeric V atoms gradually decrease while condensed polymeric V atoms increase over time. Dashed line marks ~10 ns when over 70% of V atoms have formed network structures.

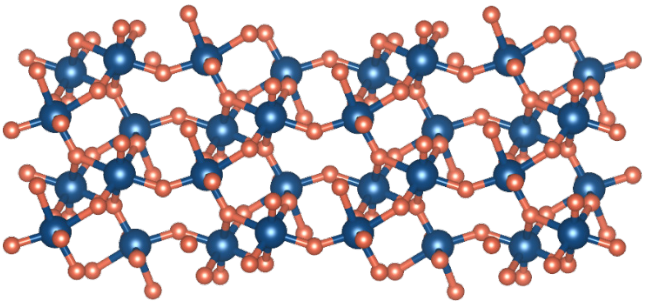

Fig. S7. Crystal structure of orthorhombic V_2O_5. Layered structure showing vanadium atoms coordinated by oxygen atoms through V-O-V bridges.

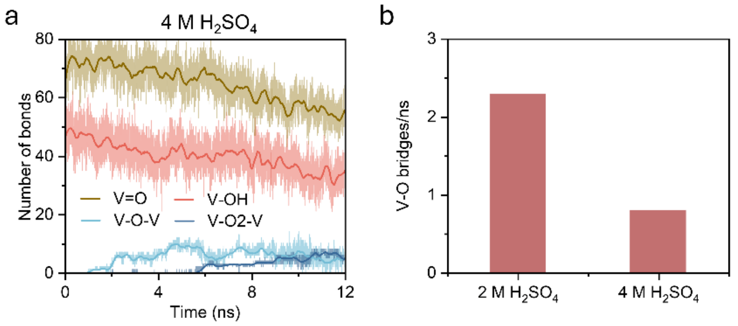

Fig. S8. Effect of sulfuric acid concentration on precipitation kinetics. (a) Evolution of coordination bond populations under 4 M H_2SO_4 conditions, showing V=O and V-OH bonds decreasing slowly while V-O-V and V-O_2-V bridging bonds increase at a significantly reduced rate compared to 2 M conditions. (b) Bridge formation rates (V-O-V + V-O_2-V per nanosecond) at 2 M and 4 M H_2SO_4, demonstrating approximately threefold suppression of transformation at higher acid concentration.

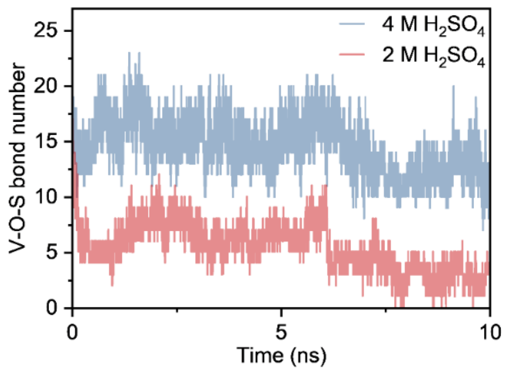

Fig. S9. Effect of sulfuric acid concentration on V-O-S coordination showing evolution of V-O-S bond numbers comparing 2 M and 4 M H_2SO_4 systems, demonstrating increased sulfate coordination at higher acid concentration.

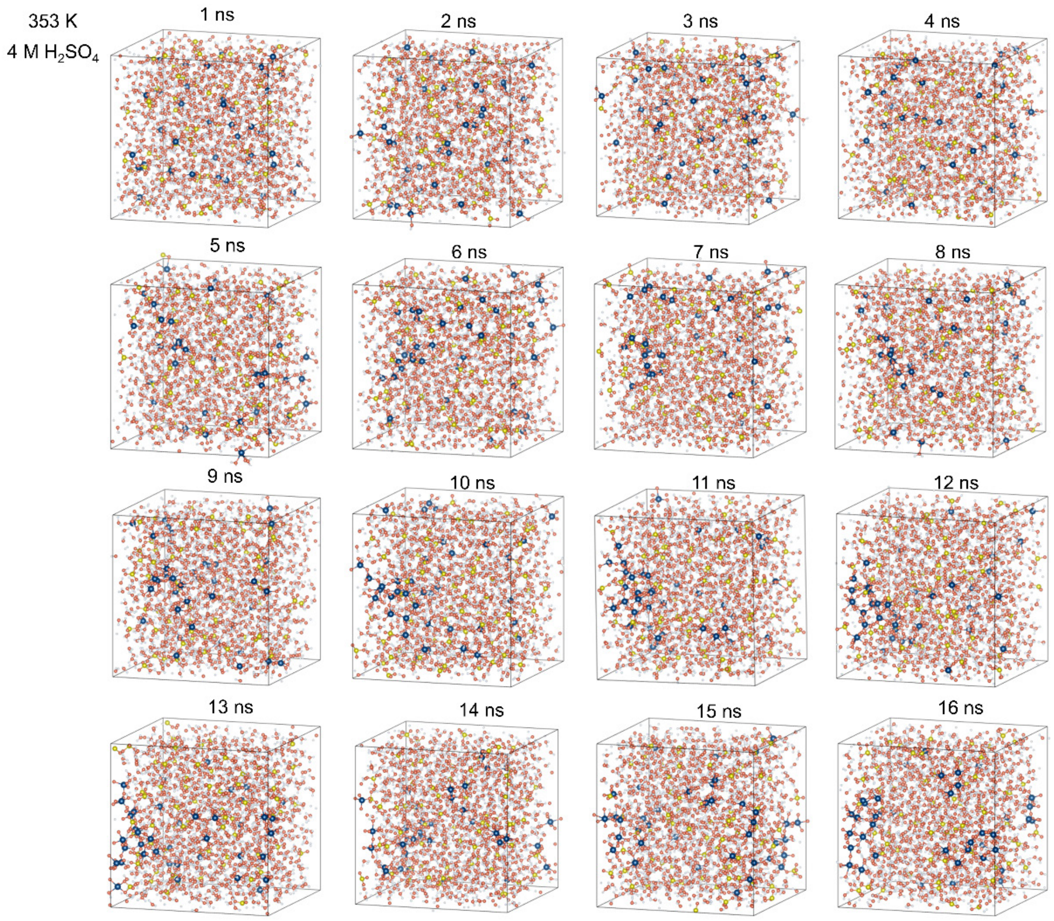

Fig. S10. Structural evolution snapshots for 4 M H_2SO_4 system at 353 K showing configurations at 1 ns interval from 1-16 ns, with reduced aggregate size and density compared to lower acid concentration.

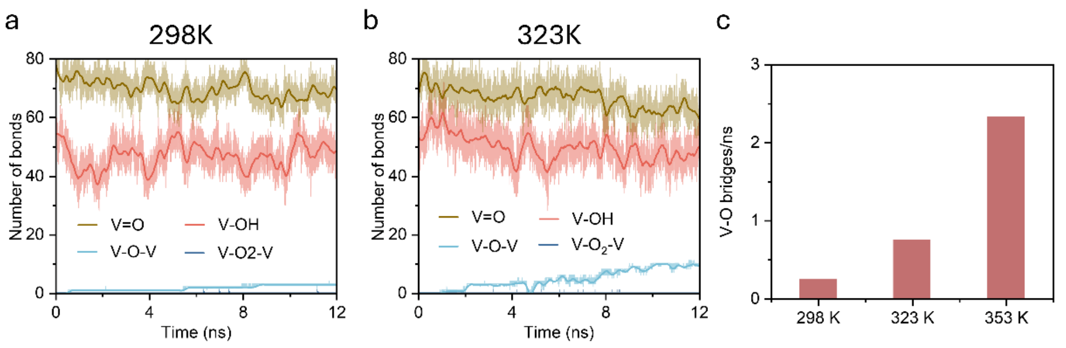

Fig. S11. Effect of temperature on precipitation kinetics. (a, b) Evolution of coordination bond populations at (a) 298 K and (b) 323 K, showing temperature-dependent rates of V=O and V-OH bond decrease and V-O-V and V-O_2-V bridge formation. (c) Bridge formation rates (V-O-V + V-O_2-V bonds per nanosecond) at different temperatures, demonstrating nearly tenfold acceleration from 298 K (0.25 bridges/ns) to 353 K (2.33 bridges/ns).

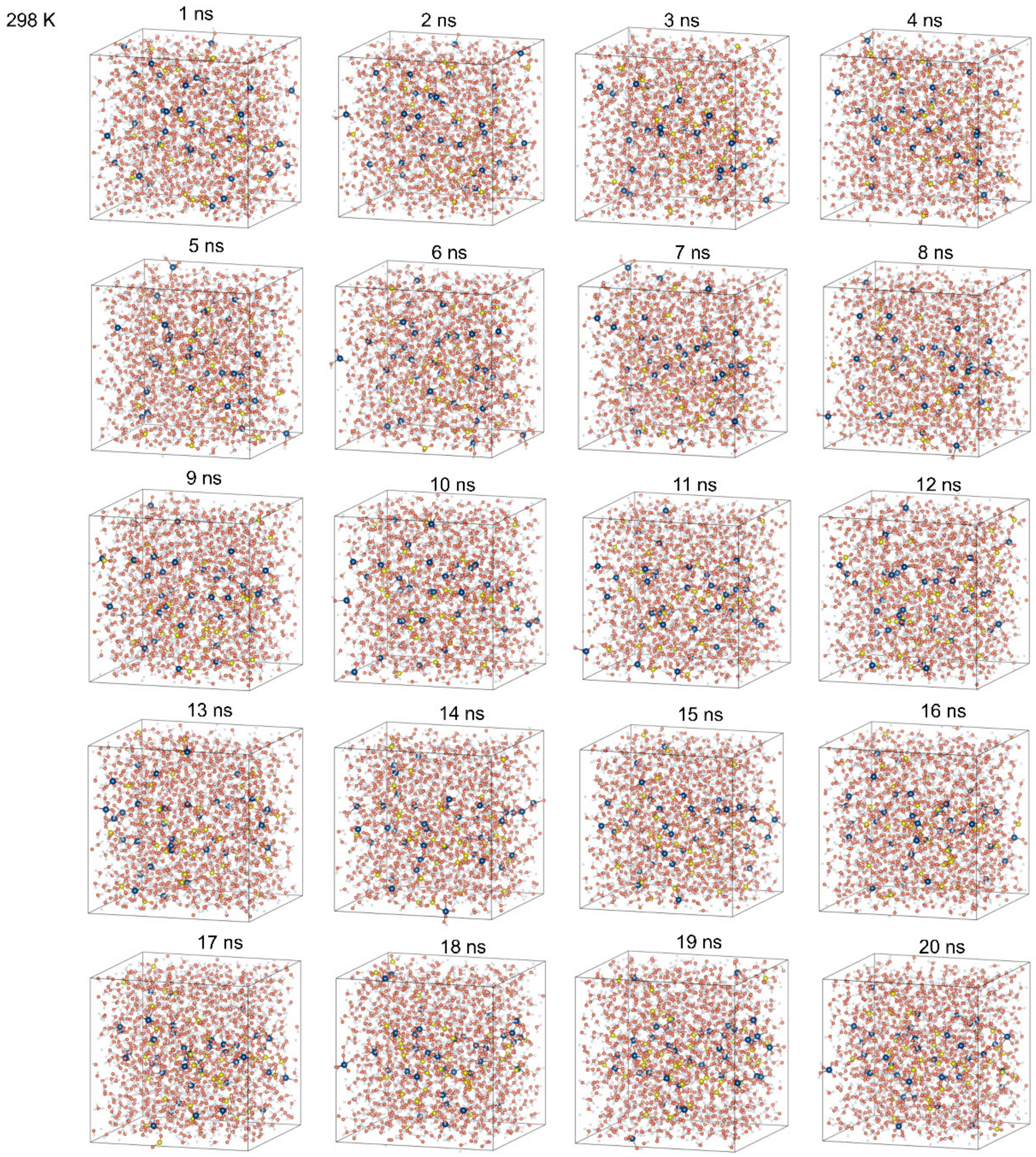

Fig. S12. Structural evolution snapshots at 298 K showing configurations at 1 ns interval from 1-20 ns, with vanadium species remaining primarily as small oligomers and large-scale network formation hindered.

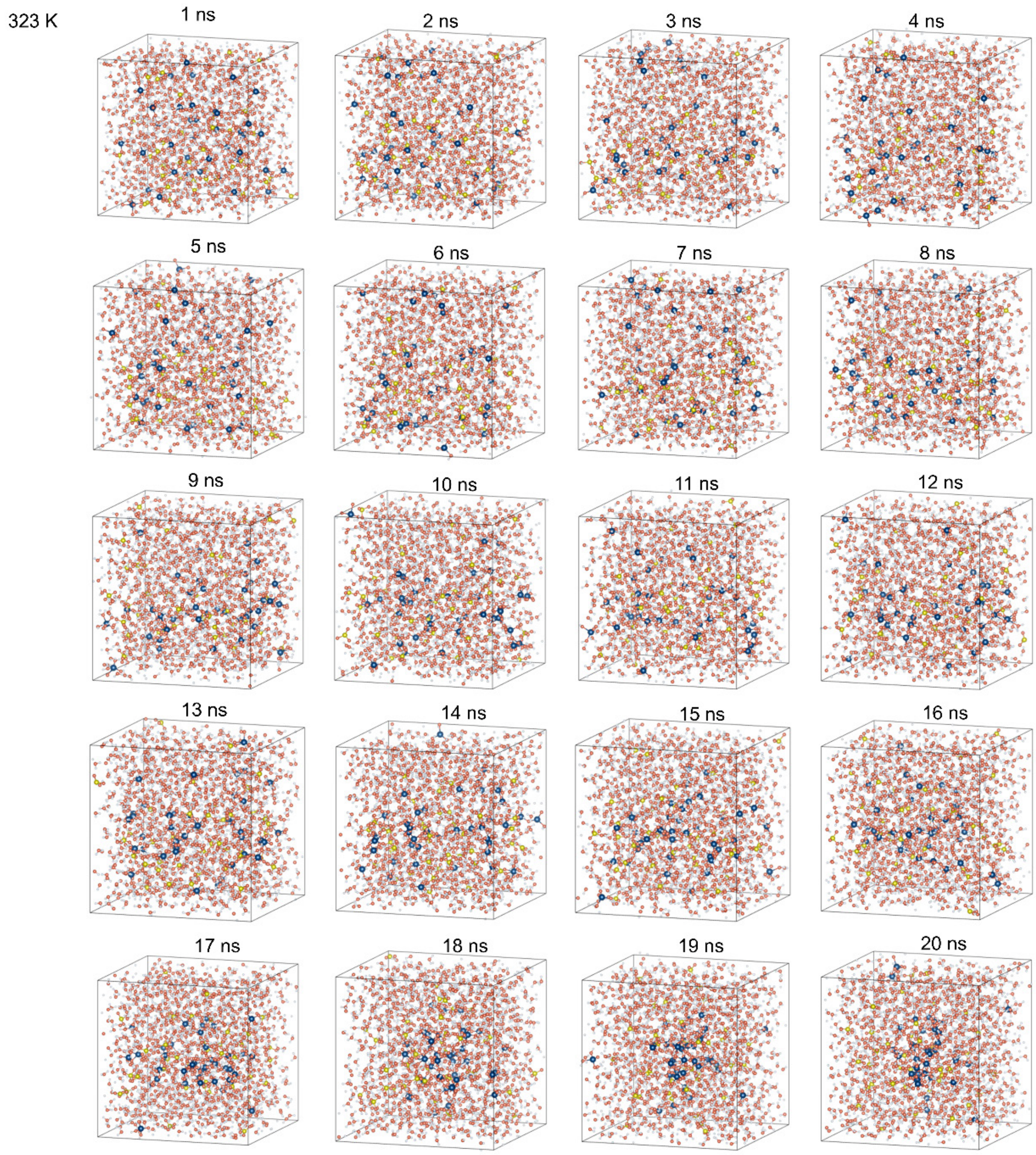

Fig. S13. Structural evolution snapshots at 323 K showing configurations at 1 ns interval from 1-20 ns, with intermediate precipitation degree and network complexity supporting temperature as a key parameter controlling precipitation kinetics.

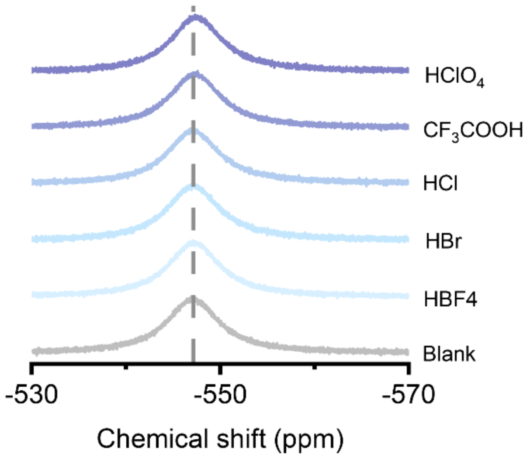

Fig. S14. ^{51}V NMR characterization of outer-sphere coordinating anions showing chemical shift spectra for HBF_4, HBr, HCl, CF_3COOH, and $HClO_4$ additives with minimal shift compared to blank sample, confirming these anions do not form stable inner-sphere coordination consistent with their limited inhibition effectiveness.

Table S1. System Composition for DPMD Simulations

System	Component	Number	Concentration (M)
System 1<br>2 M V(V) – 2 M H_2SO_4	VO_2^+	40	2 M
	HSO_4^-	40	2 M
	H_2O	1040	-
	Total atoms	3480	
System 2<br>2 M V(V) – 4 M H_2SO_4	VO_2^+	40	2 M
	HSO_4^-	80	4 M
	H_3O^+	40	2 M
	H_2O	900	
	Total atoms	3460	

References

1. T. D. Kühne, M. Iannuzzi, M. Del Ben, V. V. Rybkin, P. Seewald, F. Stein, T. Laino, R. Z. Khaliullin, O. Schütt, F. Schiffmann, D. Golze, J. Wilhelm, S. Chulkov, M. H. Bani-Hashemian, V. Weber, U. Borštnik, M. Taillefumier, A. S. Jakobovits, A. Lazzaro, H. Pabst, T. Müller, R. Schade, M. Guidon, S. Andermatt, N. Holmberg, G. K. Schenter, A. Hehn, A. Bussy, F. Belleflamme, G. Tabacchi, A. Glöß, M. Lass, I. Bethune, C. J. Mundy, C. Plessl, M. Watkins, J. VandeVondele, M. Krack and J. Hutter, The Journal of Chemical Physics, 2020, 152, 194103.
2. J. P. Perdew, K. Burke and M. Ernzerhof, Physical Review Letters, 1996, 77, 3865-3868.
3. M. Krack, Theoretical Chemistry Accounts, 2005, 114, 145-152.
4. J. VandeVondele and J. Hutter, The Journal of Chemical Physics, 2007, 127, 114105.
5. D. Zhang, H. Bi, F.-Z. Dai, W. Jiang, X. Liu, L. Zhang and H. Wang, npj Computational Materials, 2024, 10, 94.
6. Y. Zhang, H. Wang, W. Chen, J. Zeng, L. Zhang, H. Wang and W. E, Computer Physics Communications, 2020, 253, 107206.
7. L. Martínez, R. Andrade, E. G. Birgin and J. M. Martínez, Journal of Computational Chemistry, 2009, 30, 2157-2164.
8. A. P. Thompson, H. M. Aktulga, R. Berger, D. S. Bolintineanu, W. M. Brown, P. S. Crozier, P. J. in 't Veld, A. Kohlmeyer, S. G. Moore, T. D. Nguyen, R. Shan, M. J. Stevens, J. Tranchida, C. Trott and S. J. Plimpton, Computer Physics Communications, 2022, 271, 108171.
9. M. Bonomi, D. Branduardi, G. Bussi, C. Camilloni, D. Provasi, P. Raiteri, D. Donadio, F. Marinelli, F. Pietrucci, R. A. Broglia and M. Parrinello, Computer Physics Communications, 2009, 180, 1961-1972.
10. M. J. Frisch, G. W. Trucks, H. B. Schlegel, G. E. Scuseria, M. A. Robb, J. R. Cheeseman, G. Scalmani, V. Barone, G. A. Petersson, H. Nakatsuji, X. Li, M. Caricato, A. V. Marenich, J. Bloino, B. G. Janesko, R. Gomperts, B. Mennucci, H. P. Hratchian, J. V. Ortiz, A. F. Izmaylov, J. L. Sonnenberg, D. Williams-Young, F. Ding, F. Lipparini, F. Egidi, J. Goings, B. Peng, A. Petrone, T. Henderson, D. Ranasinghe, V. G. Zakrzewski, J. Gao, N. Rega, G. Zheng, W. Liang, M. Hada, M. Ehara, K. Toyota, R. Fukuda, J. Hasegawa, M. Ishida, T. Nakajima, Y. Honda, O. Kitao, H. Nakai, T. Vreven, K. Throssell and Montgomery, 2016.
11. S. Grimme, S. Ehrlich and L. Goerigk, Journal of Computational Chemistry, 2011, 32, 1456-1465.
12. J. Tomasi, B. Mennucci and E. Cancès, Journal of Molecular Structure: THEOCHEM, 1999, 464, 211-226.
13. F. Weigend and R. Ahlrichs, Physical Chemistry Chemical Physics, 2005, 7, 3297-3305.
